# Cyber anti-intellectualism and science communication during the COVID-19 pandemic: a cross-sectional study

**DOI:** 10.3389/fpubh.2024.1491096

**Published:** 2025-01-15

**Authors:** Yan Kuang

**Affiliations:** School of Journalism and New Media, Xi’an Jiaotong University, Xi’an, China

**Keywords:** science communication, anti-intellectualism, expert trust, communication effect, chain mediation model, COVID-19

## Abstract

**Background:**

During the COVID-19 pandemic, science communication played a crucial role in disseminating accurate information and promoting scientific literacy among the public. However, the rise of anti-intellectualism on social media platforms has posed significant challenges to science, scientists, and science communication, hindering effective public engagement with scientific affairs. This study aims to explore the mechanisms through which anti-intellectualism impacts science communication on social media platforms from the perspective of communication effect theory.

**Method:**

This study employed a cross-sectional research design to conduct an online questionnaire survey of Chinese social media users from August to September 2021. The survey results were analyzed via descriptive statistics, *t*-tests, one-way ANOVA, and a chain mediation model with SPSS 26.0.

**Results:**

There were significant differences in anti-intellectualism tendency among groups of different demographic characteristics. The majority of respondents placed greater emphasis on knowledge that has practical benefits in life. Respondents’ trust in different groups of intellectuals showed significant inconsistencies, with economists and experts receiving the lowest levels of trust. Anti-intellectualism significantly and positively predicted the level of misconception of scientific and technological information, while significantly and negatively predicting individuals’ attitudes toward science communication. It further influenced respondents’ behavior in disseminating scientific and technological information through the chain mediation of scientific misconception and attitudes toward science communication.

**Conclusion:**

This research enriches the conceptual framework of anti-intellectualism across various cultural contexts, as well as the theoretical framework concerning the interaction between anti-intellectualism and science communication. The findings provide suggestions for developing strategies to enhance the effectiveness of science communication and risk communication during public emergencies.

## 1 Introduction

In recent years, with the rapid development of network information technology, social media platforms have become increasingly important in the dissemination and communication of scientific, medical, and health information (1). During global health crises like COVID-19, government agencies leveraged the benefits of rapid and convenient information dissemination through social media to communicate scientific information (e.g., virus transmission routes, symptoms, epidemic prevention measures etc.) to the public, enabling them to promptly understand the true situation of the epidemic and practical epidemic prevention methods, thereby maintaining normal medical order and social stability (2, 3). However, social media platforms have also fueled the spread of anti-intellectualism (4), resulting in numerous malicious attacks on experts and scientists on platforms such as Twitter and Weibo (5–8). This phenomenon has been a barrier to effective health and science communication amidst public health crises. For instance, previous research has identified anti-intellectualism as a crucial factor influencing public reception of expert public health directives and adherence to national epidemic prevention strategies (9–12).

The term “anti-intellectualism” was popularized in 1963 following the publication of American historian Richard Hofstadter’s book, *Anti-Intellectualism in American Life*. Unlike intellectualism, which strives for rationality, abstraction, and truth, anti-intellectualism does not constitute an “-ism” in the strict philosophical sense; rather, it is a “complex of traits” (13). To elaborate, Hofstadter argued that “As an idea, it is not a single proposition but a complex of related propositions. As an attitude, it is not usually found in a pure form but in ambivalence—a pure and unalloyed dislike of intellect or intellectuals is uncommon” (13). Nevertheless, Hofstadter attempted to define anti-intellectualism, noting that its attitudes and ideas are generally characterized by **“**resentment and suspicion of the life of the mind and of those who are considered to represent it.” (13) Since then, most scholars have conducted research on anti-intellectualism based on Hofstadter’s discourse on the subject. Daniel Rigney summarized anti-intellectualism into three components: anti-rationalism, unreflexive instrumentalism, and anti-elitism (14). Some scholars regarded anti-intellectualism as a component of populism (15, 16), or defined it as “the generalized distrust of experts and intellectuals” (17). In conclusion, anti-intellectualism primarily refers to two interrelated components: one is the opposition to and skepticism about intellect and knowledge; the other is the suspicion and contempt towards intellectuals. And anti-intellectualism is characterized not by critical thinking against theoretical knowledge and intellectuals, but by generalized questioning the information provided by expert groups based on one’s own emotions, experiences, and preferences (18, 19).

Due to the differences in national conditions, religion, culture, technological levels and other factors, anti-intellectualism manifests differently in various historical contexts and social realities. In China, the roots of “anti-intellectualism” can be traced back to ancient imperial power and authoritarianism (20). To stabilize feudal authoritarian monarchy, intellectual groups were frequently despised, hated, or even persecuted by the ruling elite. In contemporary China, political movements against intellectuals sparked a wave of the “uselessness of studies” in society. With social media empowering the public to express themselves more freely, anti-intellectualism has become a phenomenon of great concern on these platforms. Previous studies pointed out that specific manifestations of cyber anti-intellectualism include rebellion against authority, promotion of extremism and pursuit of direct benefits in China (8, 21).

During the COVID-19 pandemic in China, the timely dissemination of COVID-19 information to the public and the promotion of a rational understanding and scientific response to the epidemic were crucial tasks in the fight against the novel coronavirus. Experts with government backgrounds, such as Nanshan Zhong, Lanjuan Li, and Wenhong Zhang, along with both authoritative and independent media outlets, actively shared scientific information related to the epidemic on social media. However, these experts faced numerous attacks from online public opinion. For example, in July 2021, infectious disease expert Zhang Wenhong posted his thoughts on epidemic prevention on Weibo, mentioning the protective effects of vaccines and the wisdom of coexisting with the virus. The phrase “coexisting with the virus” sparked controversy in online public opinion, with a large number of netizens flooding Zhang Wenhong’s Weibo comments section to attack him with derogatory labels such as “surrenderism,” “worshiping and having blind faith in foreign things” and “traitorous infiltrator.” In addition, hashtags like “# I advise experts not to advise #” frequently appeared on the trending search lists of Weibo and Toutiao, and some netizens opted to disregard epidemic prevention guidelines in favor of believing online rumors. These events have shown the widespread anti-intellectual sentiment among Chinese social media users. This sentiment has led to blind suspicion and emotional resistance among the public toward information provided by experts, undermining the positive impact of disseminating scientific and health-related information on epidemic prevention and control efforts.

Extant research on anti-intellectualism and science communication has mainly focused on scientific consensus, trust in experts, and health information-seeking behavior. Guo et al. (22) argued that Chinese experts face a crisis of public trust and that one of the main causes of this is the ineffectiveness of science communication. Public trust in science communicators stems from their capacity to address professional challenges and the objectivity of their writings (23), and netizens who distrust experts are more likely to be misled by misinformation (24). In the wake of the COVID-19 pandemic, researchers have utilized the variable of “anti-intellectualism” to examine its impact on the public’s response to the crisis. A survey conducted by Merkley and Loewen (10) revealed that anti-intellectualism was correlated with lower levels of COVID-19 risk perception, reduced mask usage, and limited access to COVID-19-related information. Farhart et al. (25) discovered that individuals exhibiting higher tendencies of anti-intellectualism were more likely to express hesitancy regarding COVID-19 vaccination. Huang et al. (26) found that the forms of anti-intellectualism—distrust and stigmatization—affected individuals’ information seeking about SARS-CoV-2 variants in different ways.

**Figure 1:**
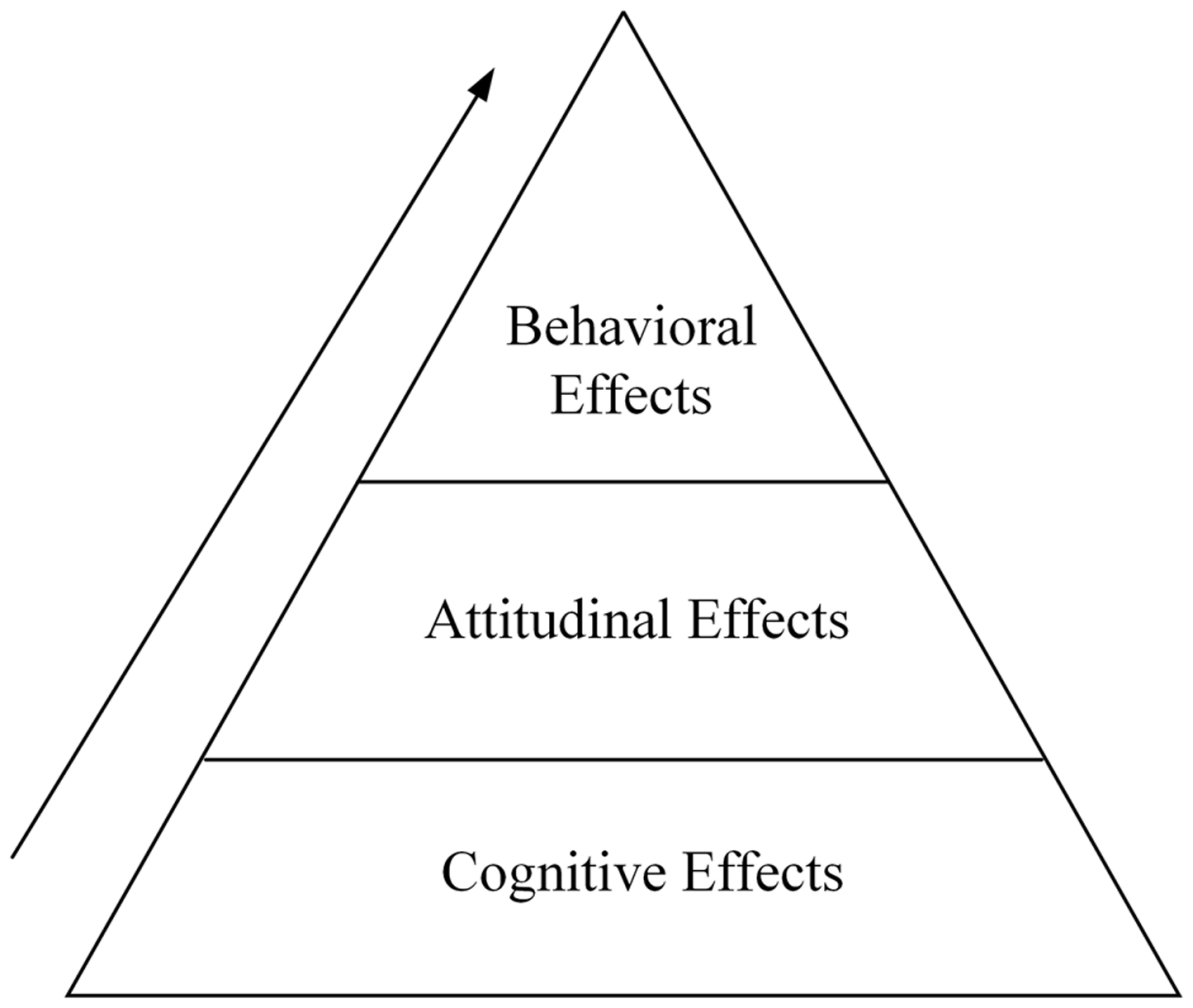
Hierarchy diagram of communication effects.

In summary, anti-intellectualism plays a key role in shaping how citizens respond to expert advice and pseudoscientific claims, as well as how they perceive public health risks. However, current research does not adequately explore the underlying mechanisms for the impact of anti-intellectualism on potential audiences of science communication. And there are fewer studies that focus on the Chinese context. Furthermore, current studies do not provide clear strategies to mitigate the effects of anti-intellectualism on science communication. Therefore, this study endeavors to investigate anti-intellectualism in Chinese social media and explore the relationship between anti-intellectualism and science communication from the perspective of the communication effect. The communication effect refers to the positive outcomes of communication activities on an audience, and specifically, resultant changes in their knowledge, emotions, attitudes, behavior, and other characteristics. It represents the degree to which these activities have achieved the communicator’s intention (27). Generally speaking, communication effects can be divided into cognitive, emotional, and behavioral levels (Figure 1). The cognitive effect refers to the impact on and changes to the audience’s knowledge system and understanding. Emotional effect refers to the impact on their attitudes and values, and changes in their actions as a direct result of the communication activities are known as behavioral effects (28). The public’s cognition, attitudes, and behaviors concerning science communication are crucial to its effectiveness. Utilizing this model, we can systematically explore the specific pathways through which anti-intellectualism impacts the science communication audiences.

Building on this background, the present study investigates Chinese social media as a field of observation, using cross-sectional survey data to analyze how anti-intellectualism influences science communication audiences in the context of COVID-19. The aim is to provide theoretical support and measures to expand the research framework of anti-intellectualism and improve the effectiveness of science communication and education.

## 2 Materials and methods

### 2.1 Research hypotheses

Based on the theoretical framework of communication effect, the study conducted a survey among Chinese users of social media platforms such as Weibo and WeChat to examine the impact of anti-intellectualism on science communication audiences from three perspectives: cognition, attitude, and behavior. By integrating the theories of anti-intellectualism and communication effect as well as prior research, the study’s hypotheses concern the degree of anti-intellectualism, scientific misconception, attitude toward science communication, and behavior related to the dissemination of scientific and technological information.

#### 2.1.1 Anti-intellectualism and science communication cognition

Social media users’ cognition of science communication involves recognizing and evaluating scientific and technological information, and distinguishing it from other types of communication (29). A correct assessment of this helps individuals distinguish between true and false knowledge, enabling them to resist the influence of pseudoscientific information. In contrast, the more individuals misinterpret scientific and technological knowledge, the lower their awareness of science communication. Anti-intellectualism relating to the public’s misconception of science is the result of the infiltration of anti-intellectual information (i.e., pseudoscience and conspiracy theories), into the scientific and technological communication environment. Anti-intellectuals reject the theories proposed by the scientific community and instead, promote irrational and subjective perceptions (30). Simultaneously, they are unwilling to heed scientific advice due to their negative attitudes toward expert communities and reject the scientific consensus (31). Based on the above analysis, this study proposes hypothesis 1:

*H1*: Anti-intellectualism significantly and positively predicts individuals’ level of scientific misconception.

#### 2.1.2 Anti-intellectualism and attitudes toward science communication

The goal of science communication is not only to popularize scientific knowledge at the cognitive level but also to recognize the essential value of science and scientists. The users’ attitudes toward science communication refer to their value judgements and emotional disposition toward science communication (32). Anti-intellectuals often harbor negative emotions toward scientific and technological knowledge, as well as expert groups. Consequently, they may exhibit a pessimistic attitude toward science communication involving scientific and technological knowledge and interactions with scientists. In addition, cognition plays an important role in attitude formation and change (33), with studies confirming this relationship in various Science and Technology (S&T) issues (32, 34). On this basis, this study proposes hypotheses 2 and 3:

*H2*: Anti-intellectualism significantly and negatively predicts individuals’ attitudes toward science communication.

*H3*: Scientific misconception plays a mediating role between anti-intellectualism and attitudes toward science communication.

#### 2.1.3 Anti-intellectualism and science communication behavior

The effect of network information dissemination is a process of accumulation, deepening, and expansion from cognition to attitude, and then to action (35). The theory of knowledge, attitude and practice (KAP) also emphasizes the logical progressive relationship between knowledge, attitude, and behavior (33). In addition, a study utilizing data from a large national survey of Canadian citizens identified a correlation between the public’s inclination toward anti-intellectualism and their information-seeking behaviors, including the consumption and discussion of COVID-19 news (10). On this basis, the study proposes the following hypotheses:

*H4*: Anti-intellectualism significantly and negatively predicts individuals’ S&T information communication behavior on social media platforms.

*H5*: Scientific misconception and attitude toward science communication play a chain mediating role between anti-intellectualism and S&T information communication behavior.

### 2.2 Data collection

This study was approved by the Ethical Review Board of the University and utilized Questionnaire Star to create electronic questionnaires for collecting data from social media users in mainland China. To ensure the overall quality of the questionnaire, this study conducted a pre-survey before the formal measurement process. A total of 100 questionnaires were distributed in the pre-survey and 95 valid questionnaires were collected. After the pre-survey, the issues identified were addressed, including revising unclear and ambiguous wording of questions, and removing items with factor loading coefficients below 0.5, before finalizing the officially implemented version of the questionnaire.

Formal participant recruitment and data collection were conducted in September 2022. Since the study focused on anti-intellectualism and science communication on social media, participants were recruited through social media (Weibo, Wechat, and Douban). The questionnaire was distributed on social media through a snowballing process. Additionally, the link to the questionnaire was posted on Weibo’s topic board and in Douban groups to expand the scope of the sample. The questionnaire was developed in Chinese. At the outset of the survey, participants were informed about the study’s purpose and procedures, and their informed consent was obtained. In order to enhance the accuracy of the survey and the representation of the population, each respondent was informed in advance that they would receive a specific amount of compensation upon completing the survey.

A total of 801 questionnaires were retrieved for the study. Questionnaires with a response time of less than 100 s and those showing a high degree of consistency in the response options of consecutive questions and incorrect responses to filtered questions were excluded, leaving a total of 563 valid questionnaires. This represented a 70.29% validity rate. Of these, there were 242 male and 321 female samples. The age of the questionnaire participants was between 20 and 60 years old, which aligns with the age distribution characteristics of the users of social media platforms. In terms of educational level, the proportion of respondents with a bachelor’s degree or higher was 79.40%, indicating a strong representation of more highly educated people, which is consistent with the specialized nature of science communication content. In terms of monthly income, respondents reported a range of RMB 2,000–10,000 (59.67%), which aligns with the average monthly disposable income of residents in 2023 (RMB 3,267) recorded by the National Bureau of Statistics, therefore, the sample is representative to a certain extent.

### 2.3 Variable measurement

To ensure the reliability and validity of the scales used in this study, those for each variable were derived or modified from established scales and literature reviews of related studies.

#### 2.3.1 Level of anti-intellectualism

Prior studies have proposed several methods to measure anti-intellectualism. Marques et al. suggested using items such as “Working on difficult intellectual problems is enjoyable and stimulating for me” to measure anti-intellectualism (36). Oliver and Rahn (37) measured anti-intellectualism with items such as “I’d rather put my trust in the wisdom of ordinary people than the opinion of experts and intellectuals” and “When it comes to really important questions, scientific facts do not help that much.” Merkley (17) assessed anti-intellectualism by examining the public’s trust in various groups of experts, such as scientists, economists, and university professors. Huang et al. (26) argued that conceptualizing anti-intellectualism solely as “distrust of experts” fails to capture its complex connotations. They proposed measuring anti-intellectualism through two dimensions: the “distrust form of anti-intellectualism” and the “stigmatization form of anti-intellectualism” (26).

As Hofstadter noted, anti-intellectualism itself is a collection of complex ideas and attitudes. In reality, in addition to the expression of absolute anti-knowledge and anti-expert, it mostly exists as a complex emotional attitude (13). Consequently, evaluating anti-intellectualism through a singular dimension oversimplifies the concept and fails to capture its nuanced meaning (17, 26).

With reference to existing measurement scales and the study of anti-intellectualism communication patterns on social media platforms, the study measured anti-intellectualism across two dimensions: ideological identification with anti-intellectualism and trust in intellectuals. The goal is to integrate the strengths of existing scales while offering a more comprehensive conceptual framework for understanding anti-intellectualism. Ultimately, the two dimensions were combined and their averages calculated to create the “Level of Anti-Intellectualism” indicator, which was used to assess the anti-intellectualism tendency of respondents.

In order to evaluate ideological identification with anti-intellectualism, this study surveyed respondents on their attitudes toward six typical anti-intellectualism stances by utilizing and modifying established scales (36–38). For example, (1) I’d rather put my trust in the wisdom of ordinary people than the opinion of experts and intellectuals; and (2) When it comes to the truly important issues, theoretical knowledge does not offer much assistance. The questionnaire used a 5-point Likert scale (1 = strongly disagree, 5 = strongly agree) to assess the extent to which respondents agreed with the above ideological views (*M* = 2.356, SD = 0.764, Cronbach’s *α* = 0.788).

The respondents’ attitudinal tendencies toward intellectuals were assessed by examining their trust in six types of expert groups as an observational variable. This was measured by asking respondents, “How much do you trust the members of the following groups?” The responses were rated on a scale from very distrustful (5) to very trustful (1), then summed and averaged to determine the level of respondents’ trust toward experts (17). The groups identified by the questionnaire consisted of experts, scientists, economists, university professors, doctors and medical professionals, and legal professionals, encompassing a broad spectrum of disciplines in the natural sciences, humanities, and social sciences. In reality, an expert may belong to more than one group, however, this study focuses on the degree of respondents’ trust in various groups, which is reflected as a consistent and stable personal tendency and differs from the trust placed in an expert within a specific context (39) (*M* = 2.362, SD = 0.560, Cronbach’s *α* = 0.858).

#### 2.3.2 Level of scientific misconception

The assessment of scientific misconception was founded on Chu’s method (29) for identifying scientific rumors. The questions were adapted from the annual “Top 10 Science Rumor Dispelling List” published by the China Association for S&T from 2019 to 2021. The list included five assertions: (1) radiation from 5G base stations affects people’s physical health, (2) taking antihypertensive drugs increases the risk of contracting the new coronavirus, (3) the less edible oil you eat, the better, (4) “0 sucrose” means no sugar, and (5) quantum fluctuation speed reading can improve learning ability. Respondents were asked to rate the above statements on a 5-point Likert scale (5 = absolutely true, 1 = absolutely false), which was used to assess their knowledge of science communication information (*M* = 2.239, SD = 0.689, Cronbach’s *α* = 0.739).

#### 2.3.3 Attitude toward science communication

Based on the scale design by Qi (32) and Gu (40), this study investigated respondents’ attitudes toward three statements, e.g., “Science communication can promote national scientific and technological progress and prosperity” and “Science communication can improve public scientific literacy.” Respondents were asked to indicate their level of agreement with the above views on a 5-point Likert scale (1 = strongly disagree, 5 = strongly agree) (*M* = 4.303, SD = 0.702, Cronbach’s *α* = 0.766).

#### 2.3.4 Science communication behavior

On social media platforms, science communication behavior includes the dimensions of browsing, discussing, liking, and sharing S&T information. Based on the scales developed by He (41) and Lan et al. (42), this study investigated respondents’ S&T information dissemination behavior, which they were asked to rate using a 5-point Likert scale (1 = never, 5 = always). The questions included (1) the frequency of using social media applications (including Wechat, Weibo, Toutiao and other apps) to browse S&T information, (2) the frequency of using social media applications to participate in discussions about S&T information, and (3) the frequency of using social media applications to forward and share S&T information (*M* = 2.785, SD = 0.676, Cronbach’s *α* = 0.666).

### 2.4 Data analysis

The questionnaires were imported into SPSS 26.0, and the data underwent descriptive statistics and correlation analysis. The *t*-test and one-way ANOVA were used to compare the levels of anti-intellectualism of different demographic characteristics. The research hypotheses were analyzed using Model 6 of the SPSS PROCESS macro program developed by Hayes (43). Bootstrap bias correction tests with 95% confidence intervals were conducted.

### 2.5 Reliability and homology bias test

Cronbach’s alpha coefficient was utilized to assess the reliability of the scale in this study. The reliability coefficients for scales with more than five items exceeded 0.7, indicating satisfactory reliability of the scale used. Due to the small number of items, a value of *α* = 0.666 for science communication behavior was deemed acceptable (44).

This study primarily examined the content validity, convergent validity, and discriminant validity of the scale. Since questions on the scale were adapted from established scales and the questionnaire content was adjusted based on expert opinions, its content validity was deemed to be suitable for the study. The Kaiser-Meyer-Olkin (KMO) coefficient of the scale was 0.843, and the chi-square value of Bartlett’s sphericity test was 4934.870 (df = 253, *p* < 0.001). The factor loading coefficients of each question after rotation, obtained through validated factor analysis, were all greater than 0.5. The Composite Reliability (CR) of all latent variables exceeded 0.7, and the Average Variance Extracted (AVE) values were above 0.4, indicating acceptable convergent validity (45). The correlation coefficients between variables were all below 0.75, and the square root of the AVE values of the variables exceeded the correlation coefficients between the variable and other variables, demonstrating good discriminant validity.

Homoscedasticity bias refers to the phenomenon where data from each measured variable can create an appearance of correlation between variables that are actually uncorrelated when the data from each measured variable are sourced from the same individual. Based on this, the study conducted the Harman one-factor test (46), which showed that the variance before rotation of the first factor was 25.008% (less than 50%). Meanwhile, the results of the complete multicollinearity test demonstrated that the VIF values of all factors were between 1.046 and 1.478 (less than 3.3). The combination of these two tests suggested that the issue of homoscedasticity bias was not significant in the data obtained here.

## 3 Results

### 3.1 Characterization of levels of anti-intellectualism among respondents

In this survey, the mean score of respondents’ “level of anti-intellectualism” was 2.359 (range 1–5). The mean of the “level of identification with anti-intellectualism” was 2.356 (range 1–5). The mean for “level of trust in intellectuals” was 2.362 (range 1–5).

As shown in Table 1, respondents’ identification with anti-intellectualism ideological views was distributed between “somewhat disagree” and “not sure,” with some views leaning toward neutrality. Specifically, the highest level of agreement was with the view that “Only knowledge that has a practical use in life is worth learning,” which indicates a tendency toward instrumentalism in knowledge acquisition. Specifically, 13.32% of the respondents indicated that they “strongly agree” and 27.53% indicated that they “basically agree,” accounting for a total of 40.85%. Another 12.43% indicated that they were “not sure,” while 46.71% indicated that they “somewhat disagree” or “strongly disagree.” In addition, respondents showed a higher agreement with the statements that “Experts are merely vested interests, distant from the lives of ordinary people” and “Intellectuals have been ‘co-opted’ by special interest groups and have mostly become spokespeople for the rich, the powerful, and interest groups,” indicating a biased perception within the expert community.

**Table 1:** Respondents’ identification with anti-intellectualism.

Anti-intellectual views	Mean value	Standard deviation
Knowledge that has a practical use in life is worth learning.	2.90	1.336
Experts are merely vested interests, distant from the lives of ordinary people.	2.52	1.087
Intellectuals have been “co-opted” by special interest groups and have mostly become spokespeople for the rich, the powerful, and interest groups.	2.41	1.073
Our lives are often influenced by conspiracies devised in clandestine locations.	2.21	1.100
I’d rather put my trust in the wisdom of ordinary people than the opinion of experts and intellectuals.	2.07	0.906
When it comes to the truly important issues, theoretical knowledge does not offer much assistance.	2.01	1.038

**Figure 2:**
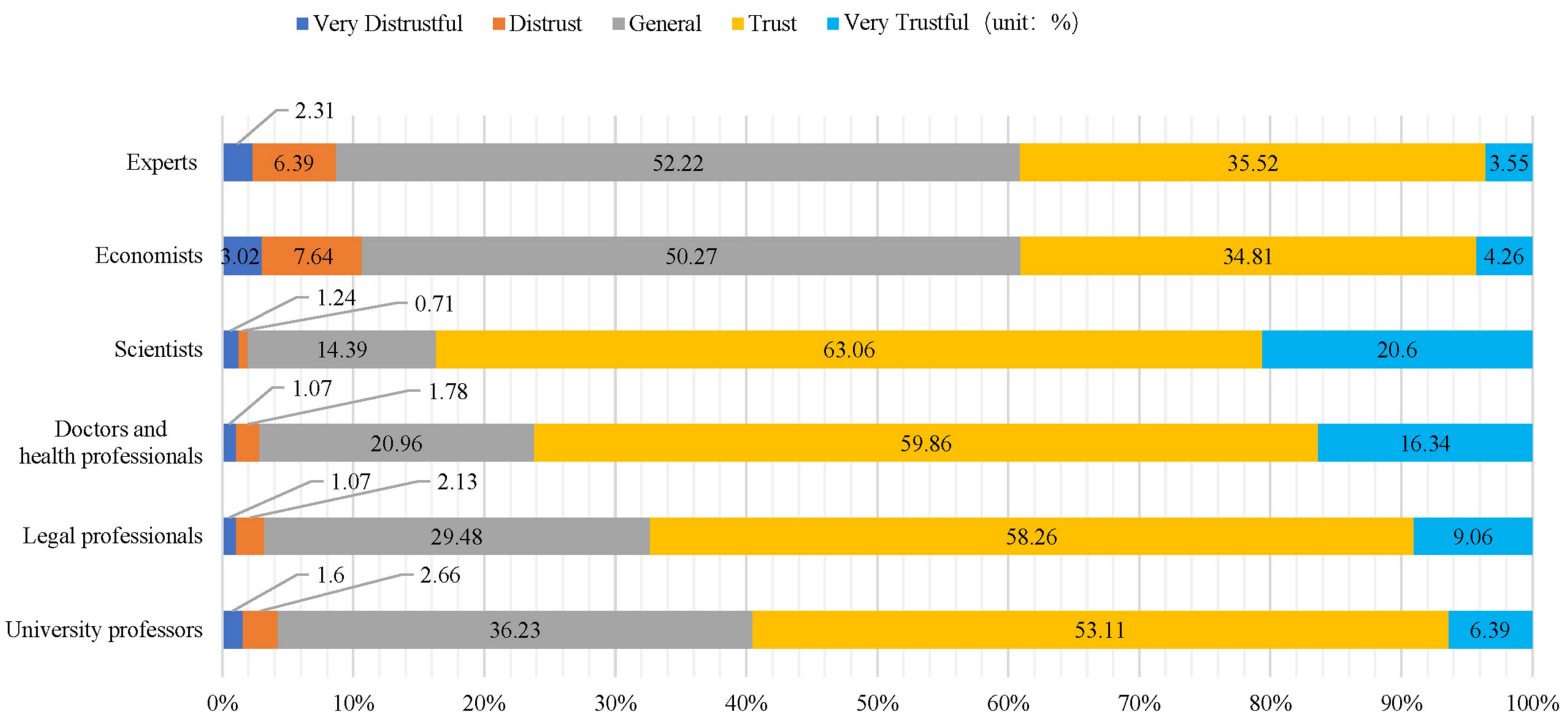
Respondents’ trust in intellectuals.

As illustrated in Figure 2, respondents exhibited the highest level of trust in scientists (*M* = 1.99, SD = 0.701), followed by doctors and health professionals (*M* = 2.11, SD = 0.727), and legal professionals (*M* = 2.28, SD = 0.700) and university professors (*M* = 2.40, SD = 0.720). Respondents had the least trust in experts (*M* = 2.68, SD = 0.745) and economists (*M* = 2.70, SD = 0.793). Specifically, the trust levels for scientists and medical professionals stood at 83.66 and 76.2%, respectively, while only 39.07% of respondents rated their attitudes toward economists and experts as “very trusting” or “trusting.”

There was a significant positive correlation between identification with an anti-intellectualism viewpoint and trust in intellectuals, with a correlation coefficient of 0.290 (*p* < 0.01). The level of anti-intellectualism in groups with different demographic characteristics is shown in Table 2.

**Table 2:** Level of anti-intellectualism in different demographic groups.

Variables	Classification	Number of respondents	Ideological identification with anti-intellectualism		Trust in intellectuals	
		n	Mean ± SD	t/F	Mean ± SD	t/F
Sex				−0.996		1.247
	Male	242	2.319 ± 0.793		2.395 ± 0.622	
	Female	321	2.384 ± 0.742		2.336 ± 0.507	
Age				3.521**		3.605**
	Under 20	18	2.500 ± 0.739		2.278 ± 0.478	
	20–29	224	2.324 ± 0.706		2.313 ± 0.483	
	30–39	169	2.185 ± 0.680		2.310 ± 0.524	
	40–49	49	2.568 ± 0.764		2.350 ± 0.517	
	50–59	61	2.522 ± 0.836		2.434 ± 0.642	
	Over 60	42	2.663 ± 1.070		2.774 ± 0.825	
Educational attainment (including current education)				3.863**		5.415***
	High school and below	50	2.720 ± 0.660		2.637 ± 0.691	
	Junior college	66	2.599 ± 0.882		2.530 ± 0.641	
	Undergraduate	199	2.346 ± 0.782		2.297 ± 0.517	
	Master’s degree or above	248	2.235 ± 0.668		2.312 ± 0.517	
Monthly income				3.746**		3.843**
	2,000 yuan and below	142	2.339 ± 0.701		2.306 ± 0.510	
	2,001–5,000 yuan	171	2.525 ± 0.790		2.470 ± 0.549	
	5,001–8,000 yuan	104	2.305 ± 0.733		2.373 ± 0.561	
	8,001–10,000 yuan	61	2.260 ± 0.741		2.391 ± 0.686	
	More than 10,000 yuan	85	2.175 ± 0.816		2.200 ± 0.521	

The independent samples *t*-test was used to compare means between two groups, and one-way ANOVA was used for three or more groups. * Indicates *p* < 0.05 ** indicates *p* < 0.01 *** indicates *p* < 0.001.

In terms of demographic characteristics, there was no significant difference in the level of anti-intellectualism between men and women. From the perspective of age, the prevalence of an anti-intellectualism viewpoint was significantly lower in the 20–39 age group compared to younger and older age groups. Regarding the level of trust in intellectuals, the older the age group, the lower the level of trust in intellectuals.

In terms of educational level, the higher the level of education, the lower the identification with an anti-intellectual viewpoint. In terms of attitudes toward intellectuals, respondents with a bachelor’s degree had the highest level of trust in intellectuals, followed by those with a master’s degree or higher. Individuals with a junior college or high school education or below exhibited a lower level of trust in intellectuals.

In terms of monthly income, the pattern of “low at both ends and high in the middle” was apparent: the group earning 2,001–5,000 yuan per month showed significantly higher levels of distrust toward intellectual groups and identification with anti-intellectualism compared to those earning 2,000 yuan or less and 5,001 yuan or more.

### 3.2 Anti-intellectualism’s immediate effects

The results of the data analysis revealed that anti-intellectualism was a significant positive predictor of levels of scientific misconception (*β* = 0.291, *p* < 0.001) and a significant predictor of negative attitudes toward science communication (*β* = −0.395, *p* < 0.001); however, this did not have a significant effect on S&T information dissemination behavior. Hypotheses H1 and H2 were supported, but hypothesis H4 was not. The results of the regression analysis of the relevant variables are presented in Table 3.

**Table 3:** Regression results for variables related to anti-intellectualism and science communication.

Variables	Scientific misconception	Attitude toward science communication	Science communication behavior
Sex	0.154 ***	−0.039	−0.180 ***
Age	0.242 ***	−0.021	0.109 *
Educational attainment	−0.062	0.087	0.161 **
Monthly income	−0.016	−0.029	0.024
anti-intellectualism	0.291 ***	−0.395 ***	−0.076
Scientific misconception		−0.209 ***	0.029
Attitude toward science communication			0.134 **
R^2^	0.217	0.299	0.092
F	30.913 ***	39.456 ***	8.074 ***

The β values in the table represent standardized coefficients. * Indicates *p* < 0.05, ** indicates *p* < 0.01, *** indicates *p* < 0.001.

### 3.3 Mediation effect test

**Figure 3:**
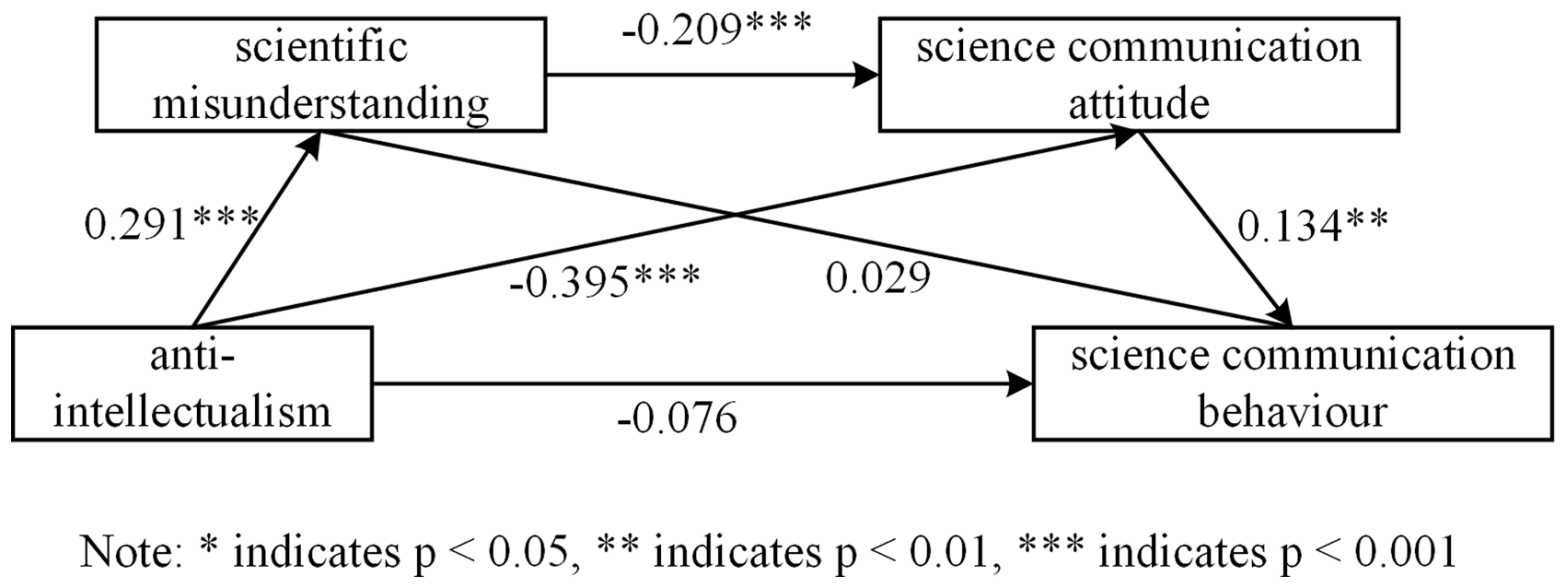
Chained intermediary model diagram.

In this study, bootstrap mediation effect tests with 95% confidence intervals were conducted, controlling for gender, age, education, and monthly income of the sample. The sample size was set at 5,000 to analyze the indirect effects of scientific misconceptions and attitudes toward science communication on anti-intellectualism and S&T information dissemination behavior. If the confidence interval does not include 0, it indicates that the indirect effect is significant. This model is presented in Figure 3. Results showed that scientific misconceptions significantly negatively predict science communication attitudes (*β* = −0.209, *p* < 0.001), and science communication attitudes significantly positively predicted S&T information dissemination behavior (*β* = 0.134, *p* < 0.01).

In the mediation model, the path coefficients of the three mediation effects of anti-intellectualism → scientific misconception → science communication attitude, anti-intellectualism → science communication attitude → science communication behavior, and anti-intellectualism → scientific misconception → science communication attitude → science communication behavior were significant. It can be inferred from this that the chained mediation effect from anti-intellectualism to S&T information communication behavior was significant based on the joint significance test. Specific analysis results are shown in Table 4. The bootstrap test of bias correction revealed that scientific misconception mediated the relationship between anti-intellectualism and attitude toward science communication. The value of the indirect effect was −0.080, with a mediation effect share of 13%. The confidence interval was [−0.126, −0.040], and the bias correction intervals were negative, supporting hypothesis H3. Science communication attitude played a mediating role between anti-intellectualism and S&T information dissemination behavior. The indirect effect value was −0.067, with a mediation effect share of 41.36%. The confidence interval was [−0.122, −0.012], and the bias correction intervals were all negative. Scientific misconception and science communication attitudes also played a chain mediating role between anti-intellectualism and S&T information dissemination behavior. The indirect effect value was −0.010, with a mediation effect share of 6.17%. The confidence interval was [−0.020, −0.002], and the bias correction intervals were all negative. Hypothesis H5 was supported.

**Table 4:** Mediation effect test.

Intermediary process	Mediating effect	SE	Relative effect (%)	95%LLCI	95%ULCI
1.Anti-intellectualism → scientific misconception → science communication attitude	−0.080	0.022	13	−0.126	−0.040
2.Anti-intellectualism → science communication attitude → science communication behavior	−0.067	0.028	41.36	−0.122	−0.012
3.Anti-intellectualism → scientific misconception → science communication attitude →science communication behavior	−0.010	0.005	6.17	−0.020	−0.002

## 4 Discussion

### 4.1 Main findings of the study

#### 4.1.1 Differences in anti-intellectualism tendency across different demographic groups

Statistical data show that older individuals tended to have lower trust in intellectuals. This may be related to the “availability heuristic.” The availability heuristic refers to the tendency for people to make decisions based on examples or events that are easily recalled from memory (47). For older individuals with more life experience, their memories are filled with various experience-based scenarios and cases. As a result, they may be more inclined to rely on their own experiences rather than expert opinions when making decisions.

In terms of educational background, there was no positive correlation between education level and trust in intellectuals. Respondents with a bachelor’s degree tended to have higher trust in intellectuals than those with a graduate degree. This may be related to the characteristics of different stages of education. In China, undergraduate education places more emphasis on the broad dissemination of foundational knowledge and the development of well-rounded skills, while graduate education focuses more on deepening specialized knowledge and training in academic research. As a result, undergraduates are more likely to focus on the general and common characteristics of intellectuals when they encounter them, while graduate students, through their deeper academic research, are more likely to recognize the internal divisions and controversies within the academic system, which in turn affects their level of trust in the entire intellectual community.

In terms of monthly income, research finds that respondents with a monthly income of 2,001–5,000 yuan exhibited significantly higher anti-intellectualism tendencies compared to those in both lower and higher income groups. This may be related to the social roles they play in the societal context. From a sociological perspective, social roles provide a powerful analytical framework for understanding an individual’s agency and normative behavior in social participation (48). In this study, respondents earning less than 2,000 yuan were primarily students, while those earning 2,001–5,000 yuan were mostly working professionals. The latter may face significant financial pressures and high expectations for their standard of living. Such psychological responses may lead to dissatisfaction and skepticism toward the external environment, thereby fostering a stronger tendency toward anti-intellectualism.

#### 4.1.2 Respondents value “knowledge that has a practical use in life”

The highest level of agreement among the respondents in this study was with the statement “Only knowledge that has a practical use in life is worth learning,” which reflects the fact that often people prioritize “whether it is useful or not” when evaluating scientific knowledge. This cognitive tendency may restrict individuals’ evaluation of science to the confines of their own cognitive abilities, and, more importantly, it reflects the importance of the scientific research’s social impact. The social impact of scientific research refers to the societal progress generated when research outcomes are transferred to society (49). This includes the effects of research on technological advancement, higher education, government decision-making, and knowledge dissemination, among other dimensions of social impact (50). In recent years, governments and various sectors of society have increasingly expected science to clearly demonstrate its social impact and practical benefits. Social impact has become a priority and requirement in international scientific research programs (51, 52). Studies have shown that scientific information based on social impact evidence can help the public overcome pseudoscience and is crucial for advancing public health (53). Therefore, demonstrating the social impact of scientific research to the public can help them understand how scientific findings contribute to solving problems in areas such as health, the environment, and daily life, which may in turn foster positive sentiments toward science and experts.

Engaging the public in the co-creation of scientific knowledge has been identified as an important strategy for achieving social impact (51). Co-creation refers to the process in which academics and other stakeholders collaboratively explore, discover, verify, and disseminate scientific knowledge (54). Public participation in the co-creation of scientific knowledge not only provides ideas, methods, and data for scientific research, driving the development and innovation of scientific knowledge, but also encourages the public to engage in scientific discussions with an evidence-based mindset through active involvement in research and science communication practices (52). This fosters the formation of scientific literacy among the public. Additionally, the public can gradually come to appreciate the value of scientific knowledge, reducing skepticism and rejection of science, which weakens the social foundation of anti-intellectualism. Therefore, involving the public in the co-creation of knowledge may be an effective strategy in public health crisis communication.

#### 4.1.3 Trust differences among different expert groups

According to the statistical data, respondents placed the highest level of trust in scientists, doctors, and medical professionals within the natural sciences, whereas expressing a relatively low level of trust in economists and those labelled as “experts.” This survey result echoes a study conducted on Norwegian citizens, which found that Norwegians placed significantly more trust in natural scientists than in climate scientists or economists. This difference in trust was attributed to the lower level of political involvement of natural scientists (55). In China, these trust differences are not only related to the characteristics of the disciplines and the political involvement of intellectuals but also to the media image of intellectuals on social media platforms. In recent years, economists and other experts have often offered “advice” on areas closely related to the public’s real-life needs and experiences, such as housing, marriage, employment, and retirement. However, these suggestions often ignore the social realities, lack constructive value, or contradict each other. As a result, they have sparked widespread controversy on social media, leading to questions regarding the reliability and accessibility of expert knowledge, as well as the credibility of the experts themselves.

In addition, the abuse of the title “expert” by some media outlets and the misinterpretation of experts’ opinions have damaged the image of the expert community. For example, some media outlets reported that Ni, an expert from the Chinese Academy of Social Sciences, claimed that “too low housing prices are detrimental to industrial transformation and also harmful to young people’s ambitions.” This statement sparked dissatisfaction among netizens. However, the expert’s actual research conclusion emphasized that housing prices and economic development follow an “inverted U-shaped relationship,” meaning both excessively high and excessively low housing prices are problematic. Yet, some media outlets, in an effort to attract attention, subjectively selected or oversimplified the expert’s viewpoint with sensationalized headlines, leading to a loss of the expert’s credibility. As a result, some netizens and media have raised the topic of # I advise experts not to advise # to express people’s dissatisfaction with the remarks by experts and the doubts about their standards. This topic repeatedly trended on platforms like Weibo and Toutiao, further fueling the aggregation of netizens’ distrust toward experts and economists.

#### 4.1.4 Expert bias and the complexity of public health crises

Some respondents’ agreement with biased views about intellectuals may stem from their suspicion that intellectuals serve as “mission sources” in science or policy communication (55, 56), believing experts are motivated by profit. In addition, this skepticism was also linked to the complexity of the COVID-19 environment in earlier years. During epidemics, scientific research has been challenged by a massive information environment, various forms of media, and conflicting government reports (57, 58). The research conclusions and prevention recommendations from experts on COVID-19 were not always timely or accurate (59, 60). For example, there were conflicting and rapidly changing scientific findings at various stages of the COVID-19 pandemic, including constant changes in the diagnostic criteria for COVID-19 pneumonia, discharge criteria, and the source of the virus. Additionally, there were “repeated changes” and “inconsistencies” in local government policies on epidemic prevention (61). In an outbreak environment, the public expects rapid reassurance (62). If experts do not provide valid information promptly, the public may question their competence.

#### 4.1.5 Effect of anti-intellectualism on cognition, attitude and behavior of science communication audience

This study demonstrates that anti-intellectualism influences S&T information dissemination behavior through the chain mediation of scientific misconceptions and attitudes toward science communication. First, an individual’s tendency toward anti-intellectualism significantly positively predicts their level of scientific misconception and significantly negatively predicts their attitude toward science communication, which is consistent with the findings of previous studies (63). In the “post-truth” era, dominated by emotional logic and personal beliefs, social media platforms collect vast amounts of information, and people tend to rely more on intuitive sensibility than rational thinking when selecting it. As a result, pseudoscientific misinformation, which manipulates experiences and emotions, hinders individuals’ ability to discern false scientific information accurately (64, 65). Second, scientific knowledge dimensions have been shown to influence attitudes and trust in science (66). This study confirms that scientific knowledge dimensions can influence attitudes toward science communication. Finally, having a positive attitude toward science communication makes individuals more willing to explore and share scientific and technological information. According to the theories of KAP, together with the theory of communication effects, the change in human behavior is divided into three consecutive processes: acquiring knowledge, forming beliefs, and shaping behavior. Additionally, there is a process of action from cognition and attitudes to behavior, in which communication activities influence the audience. Anti-intellectualism initially impacts the public’s cognition, resulting in a misinterpretation of scientific knowledge. Subsequently, it influences the public’s attitude, diminishing their positive perception of the importance of scientific communication. This leads to behavioral changes, decreasing the public’s engagement in browsing, discussing, and sharing S&T information.

This study was conducted in September 2022, when China was still in the normalization phase of COVID-19 prevention and control, and the Chinese government was implementing more stringent measures to prevent epidemics. The results suggest that even during a public crisis, anti-intellectualism can negatively influence individuals’ behavior in disseminating S&T information through a chain of mediating effects involving scientific misconceptions and attitudes toward science communication. Therefore, the government should consider implementing alternative risk communication strategies.

### 4.2 Theoretical contribution and practical implication

In recent years, anti-intellectualism in social media has shown a tendency to spread, permeating various areas such as politics, culture, S&T, and everyday life. As Hofstadter noted, anti-intellectualism often manifests in a form characterized by contradictions and fluctuations (13). Therefore, it is essential to accurately understand the conception of anti-intellectualism in conjunction with specific discourse contexts. This study starts from the context that science communication in China is facing severe challenges from anti-intellectualism. By referencing and modifying classic scales, it measures anti-intellectualism from two aspects: the degree of ideological recognition and their level of trust in intellectuals. The study aims to explore the complex relationship between anti-intellectualism and science communication during public health crisis. Its theoretical contribution lies in enriching the conceptual framework of anti-intellectualism in different cultural contexts and, from the perspective of communication effects, revealing the mechanisms through which anti-intellectualism impacts the cognitive, attitudinal, and behavioral responses of online audiences to science communication. Based on this, the study offers the following suggestions to mitigate the negative effects of anti-intellectualism on science communication:

First, government departments should introduce supporting policies to encourage and guide researchers and research institutions to pay more attention to the social impact of scientific research. The public’s increasing emphasis on “knowledge that has a practical use in life” strongly urges the scientific community to focus more on the social impact of their research outcomes. However, at present, the attention given to the social impact of scientific research in China is still insufficient (67). Therefore, government agencies should take measures to motivate researchers to consider the social impact of their work. Specifically, departments such as the Ministry of Education can strengthen the assessment of the social impact of scientific research when evaluating academic disciplines, performance, and project applications at universities and research institutes. This assessment should not only consider the impact of research outcomes on the economy, politics, and environment but also emphasize their positive effects on public values, behaviors, and societal attitudes. In addition, the Ministry of Science and Technology and other relevant agencies should develop a detailed set of guidelines for enhancing the social impact of research outcomes, outlining strategies and providing practical references for researchers.

Second, social media platforms should design specific intervention measures to urge media outlets to conduct science reporting based on scientific evidence. The findings of this study suggest that the misuse of the title “expert” and the misinterpretation or one-sided interpretation of research findings in media discourse undermine public trust in experts. In response to this phenomenon, social media platforms should refine and regulate platform rules regarding the dissemination of S&T information. This can be achieved by establishing efficient channels for online user discussions and a sound system of traffic incentives and penalties. These measures can guide media outlets to base their science communication on scientific evidence and encourage public debate. Additionally, platforms should strengthen the monitoring and optimization of algorithms, taking prompt action to restrict the promotion and lower the ranking of trending “expert” search terms that generate controversy or contain false information. This would help prevent the spread of misinformation or one-sided views and promote a more rational expression environment on the platform.

Third, experts and science communicators need to innovate risk communication strategies and engage in bottom-up science communication during public health crises. This study found that even in times of crisis, some users still perceive experts as “spokespersons for the elites and interest groups,” and their information-seeking and dissemination behaviors are negatively influenced by anti-intellectualism. This suggests that science communication and risk communication based solely on authoritative expert positions have limited effectiveness. Therefore, experts should engage in egalitarian dialogue with the public, replacing hierarchical “expert explanations” with reasoned arguments (52). In this process, science communicators should create opportunities for scientists to engage in dialogue with the public and co-create knowledge. For example, public participation in scientific activities such as online participation in scientific decision-making, science marches, and public lectures can be organized to gather the public’s opinions and needs about science. This bottom-up approach to science communication can enhance public understanding of science and trust in experts, thereby supporting the ongoing advancement of scientific innovation (53).

In addition, considering the varying degrees of anti-intellectualism among different groups, science communicator and educator should leverage artificial intelligence, user profiling, and other intelligent media technologies to address the S&T information needs and cognitive trait of individuals across regions, industries, and age groups. Based on this analysis, the appropriate content creation direction and distribution strategy would be established to enhance the effectiveness of science communication and education.

### 4.3 Limitations and future research

Based on clarification of the conceptual connotation of anti-intellectualism, this study focused on theoretical analyses and practical investigations of the propagation of anti-intellectualism in the context of the COVID-19 pandemic and its impact on the specific manifestations of science communication. This enhances the scientific validity of research on the interrelationship between anti-intellectualism and science communication. The findings on the effects of anti-intellectualism in communication serve as a reference to optimize science communication efforts, while also advancing a more profound critique and management of anti-intellectualism. Inevitably, there are shortcomings in this study. First, the data sample size collected was limited. Future research should consider expanding the scope of the sample collection to improve the scientific rigor and representativeness of the findings. Second, this study constructed a chain mediation model to explore the mechanism of anti-intellectualism on S&T information dissemination behavior. This behavior is influenced by multiple factors, and extra variables could be included to explore the mediating and moderating role of anti-intellectualism. Third, it is difficult to rigorously prove the causal relationship between variables in cross-sectional studies. Longitudinal data could be used to better elucidate the impact of anti-intellectualism. This would enable the provision of more precise countermeasure suggestions for science communication.

## Data Availability

The raw data supporting the conclusions of this article will be made available by the authors, without undue reservation.

## References

[1] Zhang W Yuan H Zhu C Chen Q Evans R. Does citizen engagement with government social media accounts differ during the different stages of public health crises? An empirical examination of the Covid-19 pandemic. Frontiers. Public Health. (2022) 10:10. doi: 10.3389/fpubh.2022.807459, PMID: 35774579 PMC9237959

[2] Kim HM Saffer AJ Liu WL Sun JY Li YQ Zhen LC. How public health agencies break through Covid-19 conversations: a strategic network approach to public engagement. Health Commun. (2022) 37:1276–84. doi: 10.1080/10410236.2021.1886393, PMID: 33591839

[3] Ngai CSB Singh RG Lu WZ Koon AC. Grappling with the Covid-19 health crisis: content analysis of communication strategies and their effects on public engagement on social media. J Med Internet Res. (2020) 22:e21360. doi: 10.2196/21360, PMID: 32750013 PMC7446717

[4] Jungherr A Posegga O An J. Populist supporters on Reddit: a comparison of content and behavioral patterns within publics of supporters of Donald Trump and Hillary Clinton. Soc Sci Comput Rev. (2022) 40:809–30. doi: 10.1177/0894439321996130

[5] Hannan J. Trolling ourselves to death? Social media and post-truth politics. Eur J Commun. (2018) 33:214–26. doi: 10.1177/0267323118760323

[6] Kirkwood GL Payne HJ Mazer JP. Collective trolling as a form of organizational resistance: analysis of the #Justiceforbradswife twitter campaign. Commun Stud. (2019) 70:332–51. doi: 10.1080/10510974.2019.1610015

[7] Linvill DL. Addressing social media dangers within and beyond the college campus. Commun Educ. (2019) 68:371–80. doi: 10.1080/03634523.2019.1607885

[8] Guo SY Lin T Akhtar N Du JA. Covid-19, anti-intellectualism, and health communication: assessing the Chinese social media platform Sina Weibo. Healthcare Basel. (2023) 11:21. doi: 10.3390/healthcare11010121, PMID: 36611581 PMC9819196

[9] Stecula DA Kuru O Hall JK. How Trust in Experts and Media use Affect Acceptance of common anti-vaccination claims. Harvard Kennedy Sch Misinform Rev. (2020). 1:1–11. doi: 10.37016/mr-2020-007

[10] Merkley E Loewen PJ. Anti-intellectualism and the mass Public's response to the Covid-19 pandemic. Nat Hum Behav. (2021) 5:706–15. doi: 10.1038/s41562-021-01112-w, PMID: 33911228

[11] Sanchez C Dunning D. The anti-scientists Bias: the role of feelings about scientists in Covid-19 attitudes and behaviors. J Appl Soc Psychol. (2021) 51:461–73. doi: 10.1111/jasp.12748, PMID: 33821031 PMC8013646

[12] Algan Y Cohen D Davoine E Foucault M Stantcheva S. Trust in Scientists in times of pandemic: panel evidence from 12 countries. Proc Natl Acad Sci USA. (2021) 40:118. doi: 10.1073/pnas.2108576118 PMC8501808 34580225

[13] Hofstadter R. Anti-intellectualism in American life. New York: Vintage (1963).

[14] Rigney D. Three kinds of anti-intellectualism: rethinking Hofstadter. Sociol Inq. (1991) 61:434–51. doi: 10.1111/j.1475-682X.1991.tb00172.x

[15] Harris L. The next American civil war: The populist revolt against the Liberal elite. New York: St Martin's Press (2010).

[16] Brewer MD. Populism in American politics. Forum J Appl Res Con. (2016) 14:249–64. doi: 10.1515/for-2016-0021, PMID: 39220592

[17] Merkley E. Anti-intellectualism, populism, and motivated resistance to expert consensus. Public Opin Quart. (2020) 84:24–48. doi: 10.1093/poq/nfz053

[18] Eigenberger ME Sealander KA. A scale for measuring Students' anti-intellectualism. Psychol Rep. (2001) 89:387–402. doi: 10.2466/pr0.2001.89.2.387, PMID: 11783568

[19] Finley S. Interrupting history: anti-intellectualism in the George W. Bush Admin Cult Stud Crit Methodol. (2009) 9:23–30. doi: 10.1177/1532708608321398

[20] Yu YS. Chinese ideological tradition and its modern changes. Guilin: Guangxi Normal University Press (2004).

[21] Luo FL Wu YJ. The expression, means of communication and governance of network anti-intellectualism. Studies in ideological. Education. (2022) 6:95–100.

[22] Guo F Sheng XM. Crisis and reconstruction of Trust in Expertise and Experts. Stud Sci. (2016) 34:1131–6. doi: 10.16192/j.cnki.1003-2053.2016.08.002

[23] Fiske ST Dupree C. Gaining trust as well as respect in communicating to motivated audiences about science topics. Proc Natl Acad Sci USA. (2014) 111:13593–7. doi: 10.1073/pnas.1317505111, PMID: 25225372 PMC4183178

[24] Mihelj S Kondor K Stetka V. Establishing Trust in Experts during a crisis: expert trustworthiness and media use during the Covid-19 pandemic. Sci Commun. (2022) 44:292–319. doi: 10.1177/10755470221100558

[25] Farhart CE Douglas-Durham E Lunz Trujillo K Vitriol JA. Vax attacks: how conspiracy theory belief undermines vaccine support. Prog Mol Biol Transl Sci. (2022) 188:135–69. doi: 10.1016/bs.pmbts.2021.11.001, PMID: 35168741 PMC8713072

[26] Huang DT Qiu HF Yang XY. Cyber anti-intellectualism and information seeking about Sars-Cov-2 variants. Chin J Commun. (2024) 17:1–21. doi: 10.1080/17544750.2023.2169836

[27] Schramm WL Porter WE. Men, women, messages, and media: Understanding human communication. New York: Harper Row (1982).

[28] Li B. Mass communication. Beijing: Tsinghua University Press (2009).

[29] Chu YJ. Why do we fall for misinformation? Cognitive Bias and misinformation discrimination from the perspective of science communication. Journal Res. (2020) 11:127.

[30] Chen WQ. Anti-intellectual subculture of mass Media in the Populized Background. Journal Commun. (2014) 21:104–28.

[31] Motta M. The dynamics and political implications of anti-intellectualism in the United States. Am Polit Res. (2018) 46:465–98. doi: 10.1177/1532673x17719507

[32] Qi WH Qi ML. The effect path of science communication on the cognition-attitude-behavior of university students towards scientific research institutions: taking Chinese Academy of Sciences as an example. Stud Sci Popul. (2021) 16:85–94. doi: 10.19293/j.cnki.1673-8357.2021.05.010

[33] Sutherland I. Health education perspectives and Choices. London: George Allen & Unwin (1979).

[34] Altenmüller MS Wingen T Schulte A. Explaining polarized Trust in Scientists: a political stereotype-approach. Sci Commun. (2024) 46:92–115. doi: 10.1177/10755470231221770

[35] Luo Y Liu B. Research on internet information communication effects. Inf Sci. (2009) 27:1487–91.

[36] Marques MD Elphinstone B Critchley CR Eigenberger ME. A brief scale for measuring anti-intellectualism. Pers Indiv Differ. (2017) 114:167–74. doi: 10.1016/j.paid.2017.04.001

[37] Oliver JE Rahn WM. Rise of the Trumpenvolk: populism in the 2016 election. Ann Am Acad Polit. (2016) 667:189–206. doi: 10.1177/0002716216662639

[38] Wang J. Investigation and analysis of the influence of populist trend of thought on mainstream ideological identity of college students. Party Build Ideolog Educ Sch. (2015) 5:86–8.

[39] Chryssochoidis G Strada A Krystallis A. Public Trust in Institutions and Information Sources Regarding Risk Management and communication: towards integrating extant knowledge. J Risk Res. (2009) 12:137–85. doi: 10.1080/13669870802637000

[40] Gu C Feng Y. Influence of public engagement with science on scientific information literacy during the Covid-19 pandemic empirical evidence from college students in China. Sci Educ Netherlands. (2022) 31:619–33. doi: 10.1007/s11191-021-00261-8, PMID: 34483484 PMC8403520

[41] He Y. Research on influencing factors of users’ scientific communication behavior in the social media field. Dalian: University of Technology (2019).

[42] Lan X Cao JD Zou NN. Analysis of the relationship between different health risk perception and user information behavior. Med Soc. (2019) 32:110–3. doi: 10.13723/j.yxysh.2019.04.028

[43] Hayes AF. Introduction to mediation, moderation, and conditional process analysis. New York: A regression-based approach: Guilford publications (2017).

[44] Hair JF Black WC Babin BJ Anderson RE. Multivariate data analysis. New Jersey: Prentice-Hall (2009).

[45] Verhoef PC Franses PH Hoekstra JC. The effect of relational constructs on customer referrals and number of services purchased from a multiservice provider: does age of relationship matter? J Acad Mark Sci. (2002) 30:202–16. doi: 10.1177/0092070302303002

[46] Podsakoff PM Mac Kenzie SB Lee JY Podsakoff NP. Common method biases in behavioral research: a critical review of the literature and recommended remedies. J Appl Psychol. (2003) 88:879–903. doi: 10.1037/0021-9010.88.5.879, PMID: 14516251

[47] Siegrist M Arvai J. Risk perception: reflections on 40 years of research. Risk Anal. (2020) 40:2191–206. doi: 10.1111/risa.13599, PMID: 32949022

[48] Smyth L. Rethinking social roles: conflict and modern life. Sociology. (2021) 55:1211–27. doi: 10.1177/00380385211007753

[49] Reale E Avramov D Canhial K Donovan C Flecha R Holm P. A review of literature on evaluating the scientific, social and political impact of social sciences and humanities research. Res Evaluat. (2018) 27:298–308. doi: 10.1093/reseval/rvx025

[50] Lima GDR Wood T. The social impact of research in business and public administration. RAE. (2014) 54:458–63. doi: 10.1590/S0034-759020140410

[51] Aiello E Donovan C Duque E Fabrizio S Flecha R Holm P. Effective strategies that enhance the social impact of social sciences and humanities research. Evid Policy. (2021) 17:131–46. doi: 10.1332/174426420x15834126054137

[52] Soler-Gallart M, Flecha R. Researchers’. Perceptions about methodological innovations in research oriented to social impact: citizen evaluation of social impact. Int J Qual Meth. (2022) 21:21. doi: 10.1177/16094069211067654, PMID: 39698541

[53] Pulido CM Ruiz-Eugenio L Redondo-Sama G Villarejo-Carballido B. A new application of social impact in social Media for Overcoming Fake News in health. Int J Env Res Pub He. (2020) 17:430. doi: 10.3390/ijerph17072430, PMID: 32260048 PMC7177765

[54] Greenhalgh T Jackson C Shaw S Janamian T. Achieving research impact through co-creation in community-based health services: literature review and case study. Milbank Q. (2016) 94:392–429. doi: 10.1111/1468-0009.12197, PMID: 27265562 PMC4911728

[55] Schroder TB. Don't tell me what I Don't want to hear! Politicization and ideological conflict explain why citizens have lower Trust in Climate Scientists and Economists than in other natural scientists. Polit Psychol. (2023) 44:961–81. doi: 10.1111/pops.12866

[56] Lewandowsky S Oberauer K. Motivated rejection of science. Curr Dir Psychol Sci. (2016) 25:217–22. doi: 10.1177/0963721416654436

[57] Tippins E Ysseldyk R Peneycad C Anisman H. Believing in science: linking religious beliefs and identity with vaccination intentions and Trust in Science during the Covid-19 pandemic. Public Underst Sci. (2023) 32:1003–20. doi: 10.1177/09636625231174845, PMID: 37278005 PMC10247686

[58] Du XY Ou LM Zhao Y Zhang Q Li ZM. Applications of advanced analysis Technologies in Precise Governance of social media rumors. Appl Sci Basel. (2021) 11:726. doi: 10.3390/app11156726, PMID: 39659294

[59] Magarini FM Pinelli M Sinisi A Ferrari S De Fazio GL Galeazzi GM. Irrational beliefs about Covid-19: a scoping review. Int J Env Res Pub Health. (2021) 18:839. doi: 10.3390/ijerph18199839, PMID: 34639241 PMC8508358

[60] Zhang T Harrington KB Sherf EN. The errors of experts: when expertise hinders effective provision and seeking of advice and feedback. Curr Opin Psychol. (2022) 43:91–5. doi: 10.1016/j.copsyc.2021.06.011, PMID: 34329943

[61] Li MD Zhang Y Zhang F Yang F Meng SJ. Content characteristics, modes, response strategies and optimization paths of epidemic scientific information dissemination: content analysis based on popular microblogs of 10 scientists. J Intelligence. (2022) 41:133.

[62] Yang LL Prasanna R King M. Gdia: eliciting information requirements in emergency first response. Requir Eng. (2015) 20:345–62. doi: 10.1007/s00766-014-0202-2

[63] Sawyer K Rhodes H. Public engagement on genetically modified organisms: When science and citizens connect. Washington, DC: National Academies Press (2015). 26225400

[64] Lu D Hong D. Emotional contagion: research on the influencing factors of social media Users' negative emotional communication during the Covid-19 pandemic. Front Psychol. (2022) 13:13. doi: 10.3389/fpsyg.2022.931835, PMID: 35911046 PMC9337870

[65] Lukyanova GV Martyanov DS. Communication strategies of emotional engagement on social media. Proceedings of the 2021 communication strategies in digital society seminar (2021).

[66] Jin JB Chu YJ. Scientific literacy, media use and social networks: understanding public trust towards scientists. Global J Media Stud. (2015) 2:65–80. doi: 10.16602/j.gmj.20150005

[67] Xiong PX Ru N. International analysis of trends in social impact evaluation of scientific research. China Univ Sci Technol. (2023) 4:22–7. doi: 10.16209/j.cnki.cust.2023.04.023

